# The Synergistic Biologic Activity of Oleanolic and Ursolic Acids in Complex with Hydroxypropyl-γ-Cyclodextrin

**DOI:** 10.3390/molecules19044924

**Published:** 2014-04-17

**Authors:** Codruţa Soica, Camelia Oprean, Florin Borcan, Corina Danciu, Cristina Trandafirescu, Dorina Coricovac, Zorin Crăiniceanu, Cristina Adriana Dehelean, Melania Munteanu

**Affiliations:** 1 Faculty of Pharmacy, Victor Babeş University of Medicine and Pharmacy, 2nd Eftimie Murgu Sq., Timişoara 300041, Romania; E-Mails: camelia.oprean@umft.ro (C.O.); fborcan@yahoo.com (F.B.); corina.danciu@umft.ro (C.D.); trandafirescu.cristina@umft.ro (C.T.); dorinacoricovac@umft.ro (D.C.); cadehelean@umft.ro (C.A.D.); 2 Faculty of Medicine, Victor Babeş University of Medicine and Pharmacy, 2nd Eftimie Murgu Sq., Timişoara 300041, Romania; 3 Department of Clinical Laboratory and Sanitary Chemistry, “Vasile Goldis” University, 1 Feleacului Str., Arad 310396, Romania; E-Mail: anaionescuro@yahoo.com

**Keywords:** oleanolic acid, ursolic acid, cyclodextrin, DMBA, TPA, mouse model, synergism

## Abstract

Oleanolic and ursolic acids are natural triterpenic compounds with pentacyclic cholesterol-like structures which gives them very low water solubility, a significant disadvantage in terms of bioavailability. We previously reported the synthesis of inclusion complexes between these acids and cyclodextrins, as well as their *in vivo* evaluation on chemically induced skin cancer experimental models. In this study the synergistic activity of the acid mixture included inside hydroxypropyl-gamma-cyclodextrin (HPGCD) was monitored using *in vitro* tests and *in vivo* skin cancer models. The coefficient of drug interaction (CDI) was used to characterize the interactions as synergism, additivity or antagonism. Our results revealed an increased antitumor activity for the mixture of the two triterpenic acids, both single and in complex with cyclodextrin, thus proving their complementary biologic activities.

## 1. Introduction

**Figure 1:**
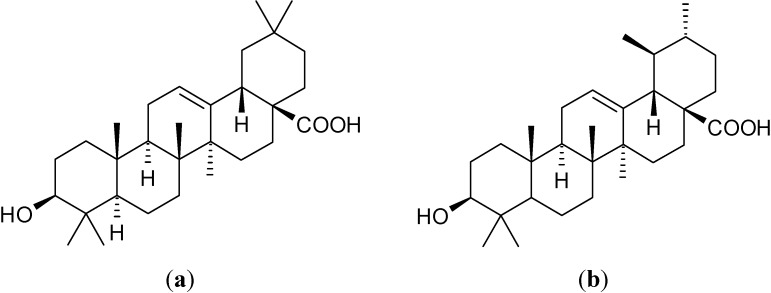
The chemical structures of: (**a**) oleanolic acid; (**b**) ursolic acid.

Oleanolic acid ((4a*S*,6a*R*,6a*S*,6b*R*,8a*R*,10*S*,12a*R*,14b*S*)-10-hydroxy-2,2,6a,6b,9,9,12a-heptamethyl-1,3,4,5,6,6a,7,8,8a,10,11,12,13,14b-tetradecahydropicene-4a-carboxylic acid, OA, Figure 1a) and ursolic acid ((1*S*,2*R*,4a*S*,6a*R*,6a*S*,6b*R*,8a*R*,10*S*,12a*R*,14b*S*)-10-hydroxy-1,2,6a,6b,9,9,12a-heptamethyl-2,3,4,5,6,6a,7,8,8a,10,11,12,13,14b-tetradecahydro-1*H*-picene-4a-carboxylic acid, UA, Figure 1b) are two pentacyclic triterpenoids widely distributed in plants [1].

OA and UA initiate an immunosuppressive effect which interferes with host parasitemia control, as revealed by a study of parasitemic levels during the acute phase of Chagas’ disease [2]. A recent report shows that an extract, containing both acids, significantly suppressed hepatitis C virus replication [3]. The inhibitory effect of OA and UA on 12-*O*-tetradecanoyl-phorbol-13-acetate (TPA) promoted tumors was *in vivo* evaluated more than 20 years ago [4]. The two acids effectively inhibit tumor promotion and initiation in mouse skin [5]. 

An interesting theory was formulated by Takada *et al.* in 2010, regarding their different capacities to block TNF-α-induced E-selectin expression; according to their study, these differences are due to conformational differences (the stable conformation of rings in UA is a twist-chair–twist-chair form, and that of OA is chair–chair), caused by the position of one methyl group (C-29) at C-19 in UA and C-20 in OA [6].

OA and UA were found as effective anti-hepatoma agents with marked anti-cancer activity [7]; they also show protective effects against H_2_O_2_-induced DNA damage in leukemic L1210, K562 and HL-60 cells as well as significant antioxidant effects [8].

More recently, a synergistic antimicrobial activity was reported for the two acids, accompanied by an immunostimulatory effect [9]. Another case of synergism was reported in 2010 in the case of oleanolic acid and insulin in STZ-induced diabetic rats [10]. Another research group reported the protective effect of a mixture of OA and UA against colon cancer [11].

Chemical carcinogenesis is a multi-stage process that begins with exposure, usually to complex mixtures of chemicals that are found in the human environment [12,13], that can be divided conceptually into four steps: tumor initiation, tumor promotion, malignant conversion and tumor progression [14]. 7,12-Dimethylbenz(a)anthracene (DMBA) is an immune-suppressor and a powerful organ-specific carcinogen used as a tumor initiator while tumor promotion can be induced by applying 12-*O*-tetradecanoyl-phorbol-13-acetate (TPA) in some models of two-stage carcinogenesis [15].

The pentacyclic triterpenes present a bulky non-polar structure and, consequently, very low water solubility. One of the most studied pathways in solving the solubility problem is the synthesis of cyclodextrin (CD) inclusion complexes [16]. 

The aim of our research was the study of the synergistic antitumor effect of the two triterpenic acids; cyclodextrin complexes of OA, UA and OA/UA mixture, respectively, were used for *in vivo* studies, in order to achieve the necessary water solubility. Based on previous studies [17] 2-hydroxypropyl-γ-cyclodextrin (HPGCD) was chosen as host molecule for the triterpenic acids and their mixture.

## 2. Results and Discussion

Viability and proliferation assay with Alamar Blue (AB) is based on the evaluation of mitochondrial activity of living cells which reduce resazurin, a dark blue compound with an intrinsic fluorescence, to resorufin, a pink and highly fluorescent compound (579 extinction/584 emission). Maximum absorbencies appear at 605 nm and 573 nm for resazurin and resorufin, respectively (according to the test manufacturer’s protocol).

**Figure 2:**
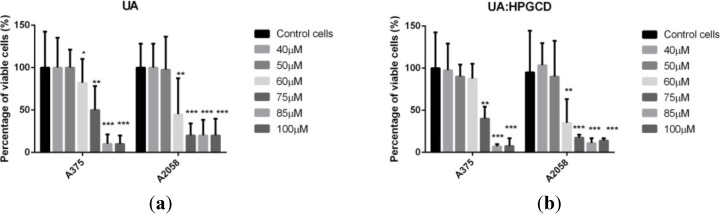
Alamar Blue assay showing the effects of (**a**) UA and (**b**) UA:HPGCD complex, respectively, on the viability of the A375 and A2058 human melanoma cell lines. Viability is expressed as percentage of viable cells compared to the control (considered as 100%). The dilution rate was 1:1000, the concentration of the stock solution was 10 mM, and UA final concentrations in the medium were 40 μM, 50μM, 60μM, 75 μM, 80μM and 100 μM.

Figure 2 shows cells’ viability in A375 and A2058 cell lines after 48 h exposure to different concentrations of ursolic acid. Ursolic acid exhibited an antiproliferative effect in a dose-dependent manner. The IC_50_ of UA in A375 and A2058 human melanoma cell lines was 75 µM and 60 µM, respectively.

At low concentrations (40 µM, 50 µM) UA did not show any cytotoxic activity neither in A375 nor in A2058 cell line while at higher concentrations (85 µM and 100 µM) a stronger antiproliferative response to UA exposure was found for the A375 cell line; in case of a medium concentration (60 and 75 µM) the strongest antiproliferative effect was found on A2058 line. Incorporation of this pentacyclic triterpene in HPGCD seems to keep up the dose-range effect of the pure active compound on both cell lines, showing the same behavior, with a slightly increased activity for certain concentrations (Figure 2b), and, except for one case, without statistical significance. The A375 human melanoma cell line stands as the exceptional case, where a significant increased activity can be noticed for the complex concentration of 85 µM (*p* = 0.046). This behavior is also valid in case of A2058 human metastaic cell line (*p* = 0.048).

**Figure 3:**
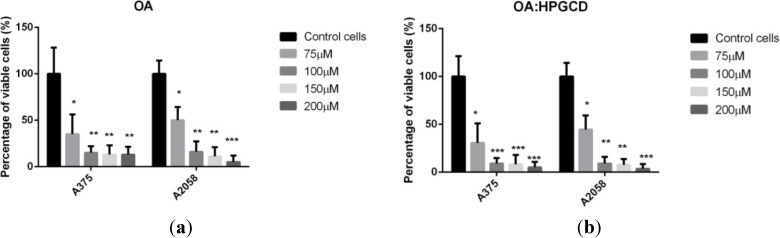
Alamar Blue assay showing the effects of (**a**) OA and (**b**) OA:HPGCD complex, respectively, on the viability of the A375 and A2058 human melanoma cell lines. Viability is expressed as percentage of viable cells compared to the control (considered as 100%). The dilution rate was 1:1000, the concentration of the stock solution was 10 mM, and the oleanolic final concentrations in the medium were 75 μM, 100μM, 150 µM and 200 μM, respectively.

As shown in Figure 3, after 48h exposure at oleanolic acid, cells viability was less than 30% of the control, for both cell lines (27% in A375 and 22% in A2058), decreasing with the concentration. Based on our previous studies on A375 human melanoma cell line, which showed a lower IC50 (between 50 and 75 µM) for the ursolic acid, we chose to use higher concentrations of oleanolic acid than the ones used in case of ursolic acid [18]. The IC_50_ of OA in A375 and A2058 human melanoma cell lines was 75 µM and 60 µM, respectively. After the incorporation of the oleanolic acid in HPGCD the same observations depicted above for the ursolic acid were valid, the cyclodextrin complexation leading to a slightly increased antiproliferative activity. Significant results were found in case of A375 cell line, starting from the concentration of 100 µM as follows: *p* = 0.047 for 100 µM; *p* = 0.046 for 150 µM; *p* = 0.043 for 200 µM. Significant results were found when the A2058 cell line and concentrations of 100 µM (*p*= 0.046) and 150 µM (*p* = 0.048) were used.

**Figure 4:**
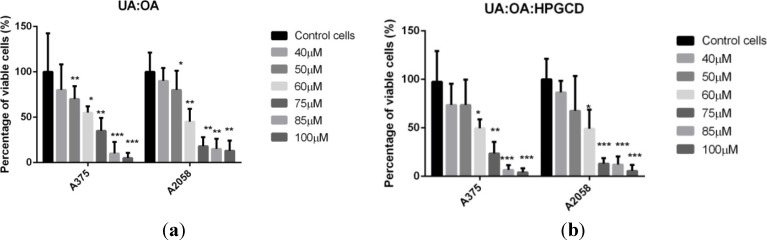
Alamar Blue assay showing the effects of (**a**) UA:OA mixture and (**b**) OA:UA:HPGCD complex, respectively, on the viability of the A375 and A2058 human melanoma cell lines. Viability is expressed as percentage of viable cells compared to the control (considered as 100%). The dilution rate was 1:1000, the concentration of the stock solution was 10 mM, and 1:1 UA:OA final concentrations in the medium were 40 μM, 50μM, 60μM, 75 μM, 80μM, and 100 μM, respectively.

The most significant results in terms of inhibiting cells viability in A375 and A2058 cell lines after 48 h were obtained in the case of exposure to different concentrations of 1:1 UA:OA mixture (Figure 4). It was a dose-dependent antiproliferative effect, where the IC_50_ of the mixture on A375 and A2058 human melanoma cell lines appear at a concentration of 60 µM. Cytotoxic activities also appeared at low concentrations (40 µM, 50 µM), but the strongest antiproliferative effect on both cells lines was achieved at 85µM and 100 µM. The use of the equimolar mixture of triterpenic acids incorporated in HPGCD led to the same behavior previously described for the pure compounds. A slightly increased activity can be seen for the A375 cell line, with no significant relevance, except for the concentration of 75 µM (*p* = 0.039). For the A2058 cell line the exception occured at the concentration of 100 µM (*p* = 0.037).

Similar results were obtained by an *in vitro* research on A2058 and A2780 cell lines, but the study only compared the individual cytotoxic activity of these triterpenic acids [19].

**Figure 5:**
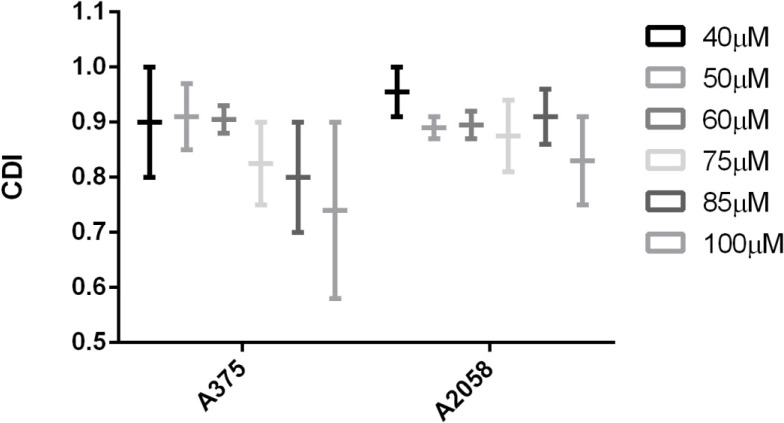
The synergistic antiproliferative effect of OA combined with UA on the growth of A375 and A2058 cell lines. CDI (coefficient of drug interaction) was calculated as follows: CDI = AB/(A × B), where AB represents the ratio between the absorbancy values of the mixture (OA + UA) and control groups while A or B are the ratio between the absorbancy values of the single agent and the control group.

**Figure 6:**
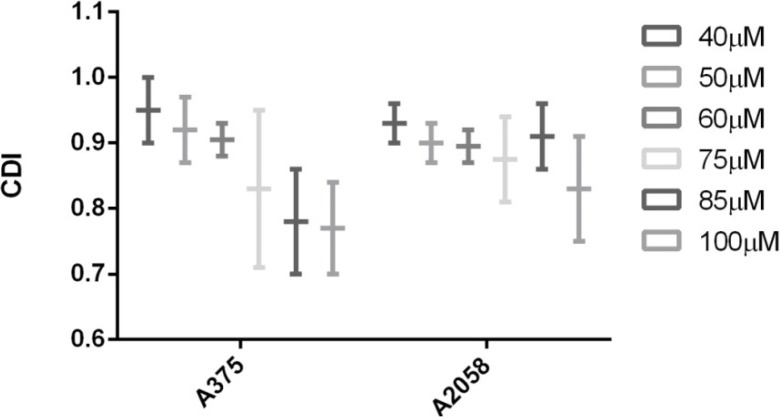
The synergistic antiproliferative effect of OA:HPGCD combined with UA: HPGCD on the growth of A375 and A2058 cell lines. CDI (coefficient of drug interaction) was calculated as follows: CDI = AB/(A × B), where AB represents the ratio between the absorbancy values of the mixture (OA + UA) and control groups while A or B are the ratio between the absorbancy values of the single agent and the control group.

Analyzing CDI values one can notice that in both cases, with or without cyclodextrin complexation, a synergistic behavior of the two triterpenic acids was recorded (CDI < 1) (Figure 5 and Figure 6); moreover, for some concentrations (e.g., 85–100 μM) applied on A375 cell line, the CDI value is very close to 0,7 which reveals a significant synergistic effect. Cyclodextrin complexation preserves this behavior and improves water solubility, leading to a higher bioavailability.

Ursolic acid was found to possess antiproliferative effect on human colon adenocarcinoma HT29 cells, human colon cancer SW480 and LoVo cells, B16 melanoma cells, human non-small cell lung cancer A549 cells [20,21,22,23]. Oleanolic acid was reported to act as antiproliferative in case of NB4 leukemic cells, human colon cancer cell lines SW480 and SW620, HT-29 colon cancer cells, HuH7 human hepatocellular carcinoma [24,25,26,27].

Inclusion of different active substances into different CDs and their effect on a wide range of cell lines have been discussed in the literature. Some groups stain that this physicochemical procedure increases the antiproliferative activity of a potent compound due to the increased cellular uptake after incorporation [28,29,30]. This kind of results were reported for betulin, albendazole, pyrazolo[3,4-*d*]pyrimidines, ferrocenyl–tamoxifen adducts [31,32,33,34]. On the other hand, other research groups reported that cyclodextrin incorporation had no influence on the antiproliferative effect of the active compound [30,35]. During our research, HPGCD encapsulation seems to have a significant effect only when used in higher concentrations (starting from 75 μM), but the observation is not widely available as described above. Given the high incidence and fast metastatic proliferation of melanoma, the use of antiproliferative compounds is mandatory; the synergistic behavior of triterpenic acid ensures smaller doses of each compound and therefore weaker side effects.

**Figure 7:**
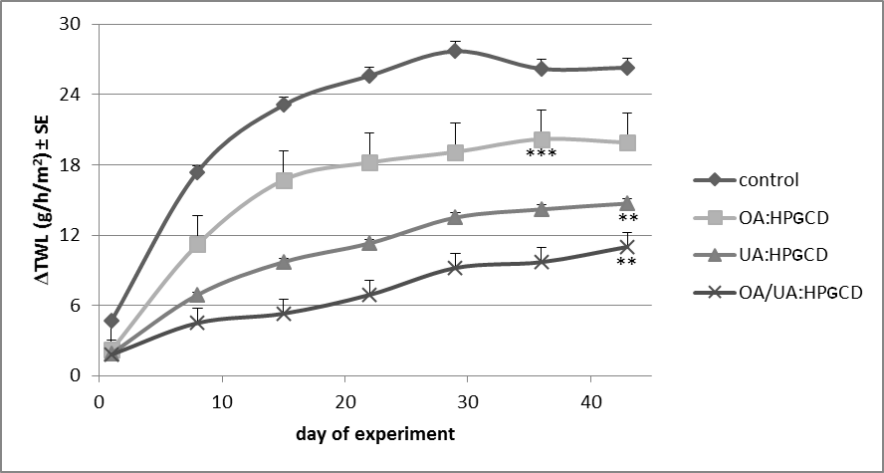
Transepidermal water loss.

**Figure 8:**
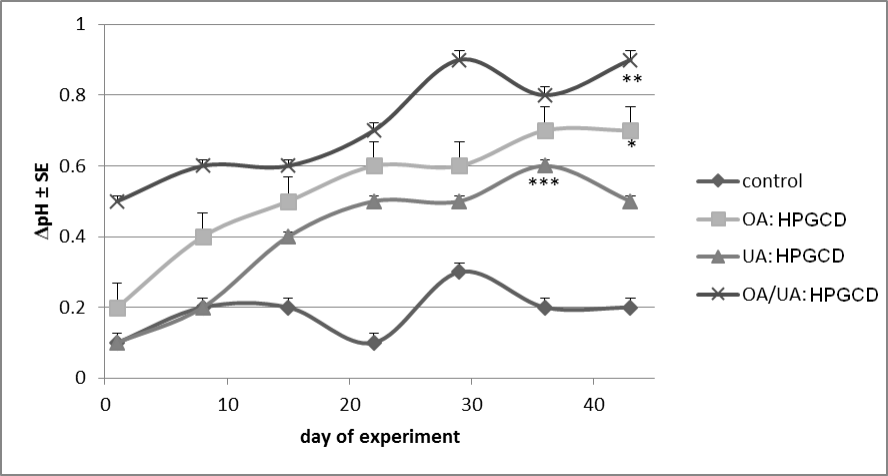
Skin-pH content.

**Figure 9:**
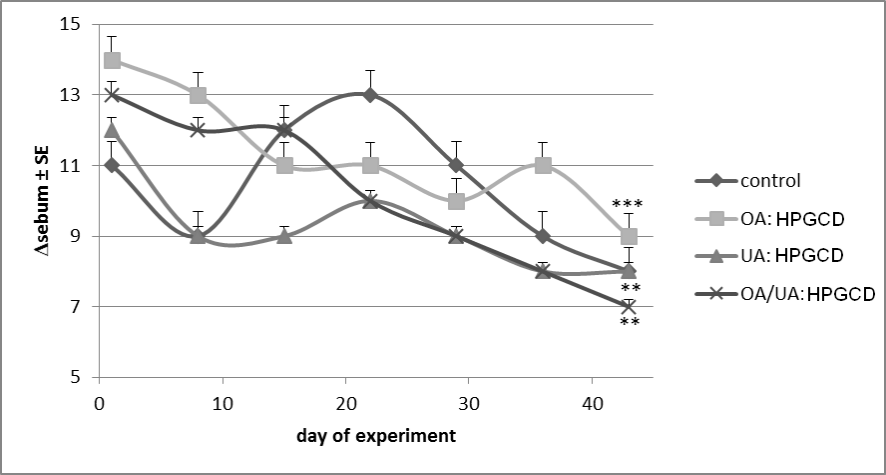
Sebum content.

**Figure 10:**
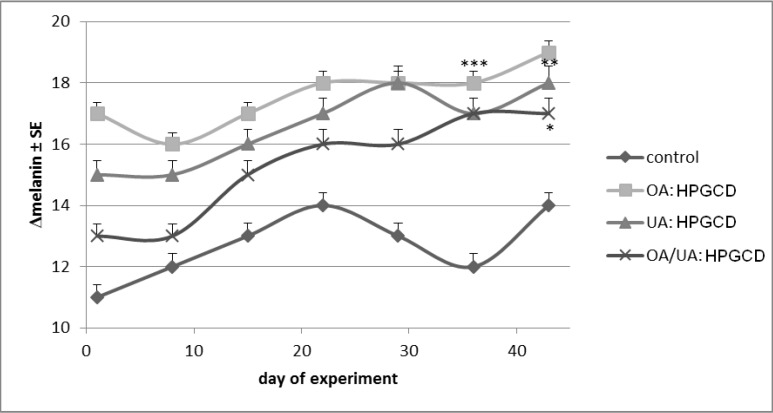
Melanin content.

**Figure 11:**
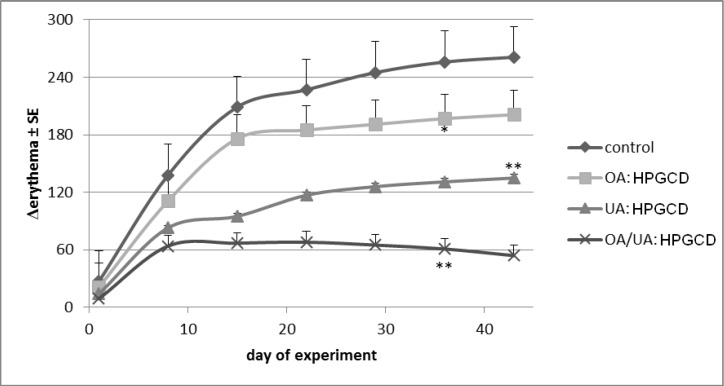
Erythema progress.

**Figure 12:**
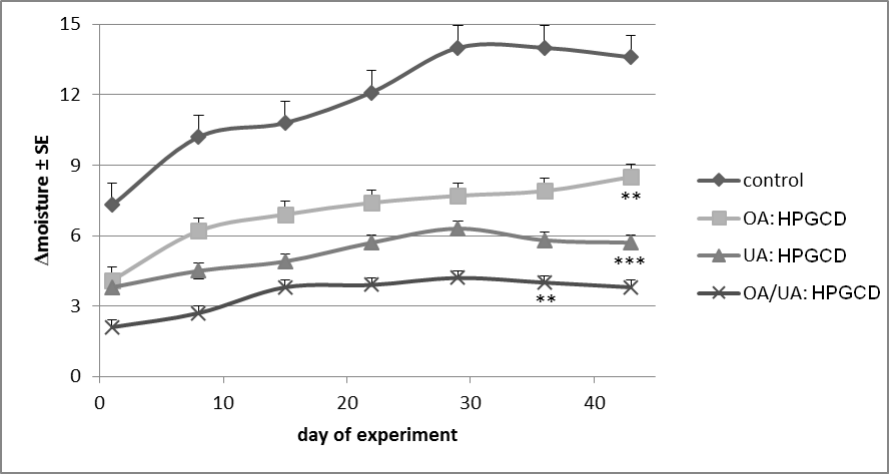
*Stratum corneum* moisture content.

The skin parameters were monitored for six weeks and data were collected in the first day of every week. The measurements were done in triplicate and are presented in Figure 7, Figure 8, Figure 9, Figure 10, Figure 11, and Figure 12 as differences between treated skin area and a blank area. The measurements of melanin and erythema serve as quantitative results regarding tumour evolution.

The TEWL measurements indicated important increases of transepidermal waterloss during the six weeks of the experiment (Figure 7). TEWL values below 10 g/h/m^2 ^ characterize a good skin condition while over 25 g/h/m^2^ values correspond to a poor skin condition [10]. In the current research, the most important change was recorded in the case of control mice (ΔTWL ~25 units/six weeks), while practically no modification was noticed in the case of 1:1:1 OA:UA:HPGCD treated mice group.

The skin-pH was approximately the same for the control mice group (maximum difference recorded from one week to another was 0.2 units). Slight increases of skin-pH values were noticed for the mice treated with cyclodextrin complexes. The most important change was seen in the case of OA/UA:HPGCD treated mice (Figure 8). Similar results were revealed in a previous study of our team [36]. 

Sebum slightly decreased values were noticed for all samples, but the changes are statistically insignificant (Figure 9).

Melanin, the most important skin pigment, suffered slight modifications for each mouse: a non linear increase of differences between 11–14 units was revealed in the control mice group, while for the other three groups the evolution of melanin difference increase was as follows: from 17 to 19 units (OA:HPGCD treated group), from 15 to 18 (UA:HPGCD treated group) and from 13 to 17 units (OA:UA:HPGCD). The most reasonable explanation of this variation would be the treatment with DMBA in the first period of experiment (Figure 10). Generally, slight and non-linear increases were obtained for all samples, but without statistical significance. 

Erythema is the most important skin parameter involved in the evaluation of drugs or chemicals irritative potential, as well as the evaluation of antimelanoma agents. An important change was recorded for the control mice group, the difference between the treated skin area and a blank area reaching more than 230 units after six weeks of experiment). By contrast, a very small difference was noticed for the mice treated with the cyclodextrin complex of the 1:1 OA:UA mixture (below 50 units after six weeks of treatment) (Figure 11).

**Figure 13:**
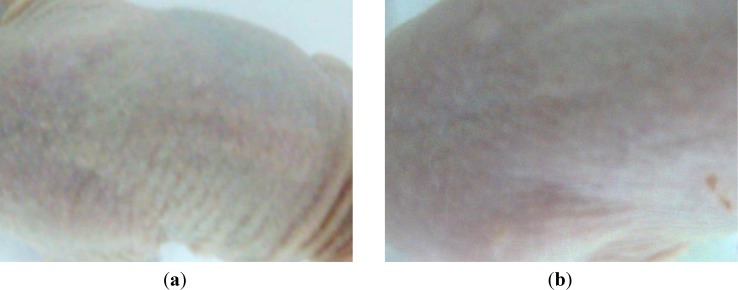
Skin macroscopical differences for: (**a**) control mouse; (**b**) OA:UA:HPGCD treated mice, after experiment.

The water loss from the *stratum corneum* appears in Figure 12 as increased differences between the exposed and unexposed skin areas. The decrease of *stratum corneum*’ moisture in the control mice group (Figure 12 and Figure 13a) reached the highest level; OA:HPGCD and UA:HPGCD treated mice groups lost more or less water from *stratum corneum* during the experiment, while the smallest difference (around two units) was noticed for the OA:UA:HPGCD treated mice group. However, the values for UA:HPGCD and OA:UA:HPGCD groups are very similar.

The MPA5 from Courage-Khazaka is a powerful tool for dermatologists, but also, for the study of any skin changes during the development of skin cancer models. TEWL and erythema increase significantly in the first week of chemically (DMBA/TPA treatment) and/or UVB induced skin cancers [36]. A recent paper dealing with the toxicity of nitrofuran-type compounds on melanoma revealed that melanin protects melanoma cells from nitrofuran-induced DNA damage [37]; however, during the current experiment, the melanin level did not fluctuate significantly. Swalwell *et al.* evaluated the role of melanin in skin cancers using human melanoma cells; they found that skin pigment prevents mitochondrial superoxide production and mitochondrial DNA damage, but does not appear to prevent cytosolic oxidative stress [38]. In a 15 weeks-experiment, Cerga *et al.* reported that the level of TEWL increased two times less for skin cancer C57BL/6j mice models treated with OA or UA—cyclodextrin complexes than the level recorded in the control group [15].

The evolution of skin surface pH in melanoma, non-melanoma skin cancers and other skin diseases was rarely assessed. J. Liu *et al.* noticed no modification of this parameter in volunteers with vitiligo [39]. Differences of skin surface pH depending on Fitzpatrick types were reported by Gunathilake *et al.*; subjects with type IV–V skin, with increased epidermal lipid content and lamellar body secretion, have more acidic *stratum corneum* surface pH [40]. Elevated pH values interfere with both permeability barrier homeostasis and *stratum corneum* integrity leading to an increased activity of serine proteases, responsible of normal desquamation [41].

In previous studies, the moisture of *stratum corneum* was used to evaluate the skin photoaging [42] or the hydration potential of UV protection creams [43]. In a 2010 US patent the hydration increase of the skin treated with a cosmetic product is attributed to ursolic acid [44]. Pentacyclic triterpenoids improve epidermal barrier function and induce collagen production thus modifying the parameters of skin [45].

## 3. Experimental

OA and UA (analytical purity) were purchased from Fluka (Sigma Aldrich, Steinheim, Germany), HPGCD was obtained from Cyclolab (Budapest, Hungary). DMBA and TPA were purchased from Sigma-Aldrich (Taufkirchen, Germany), and ethanol from Chimopar (Bucharest, Romania). The mice were obtained from Charles River Laboratories (Sulzfeld, Germany).

### 3.1. Alamar Blue in Vitro Analysis

A375 human melanoma and A2058 human metastatic melanoma (Sigma-Aldrich, Bucharest, Romania) were cultured in DMEM containing 10% FCS (fetal bovine serum, PromoCell, Heidelberg, Germany), 1% penicillin-streptomycin (Pen/Strep, 10,000 IU/mL; PromoCell) and 1% glutamine (PromoCell). Cells were maintained at an atmosphere of 5% CO_2_ at 37 °C.

The cell lines (ECACC; Sigma Aldrich origin Japan stored UK) was seeded onto a 96-well microplate (5,000 cells/plate) and attached to the bottom of the well overnight. After 24 h, 150μL of new medium containing Dulbecco’s Modified Eagle’s Medium supplemented with 10% fetal calf serum, 1% penicillin/streptomycin mixture and 1% L-glutamine and the tested substances were added and incubated for 48 h. UA, UA:HPGCD, UA:OA, UA:OA:HPGCD were added in the concentrations of 40 μM, 50 μM, 60 μM, 75 μM, 80 μM and 100 μM. Based on our previous results we have chosen to test for OA, OA:HPGCD respectively higher concentrations, namely 75 μM, 100 μM, 150 μM, 200 μM [18]. The CD alone was tested in the same range of concentrations but didn’t have any effect on the proliferation of the cells. After 48 h, 15 μL of the Alamar Blue solution was added and the cells were incubated for 4 h at 37 °C. Finally, the samples were spectrophotometrically analyzed at 570 nm, 600 nm respectively, using a microplate reader; wells with untreated cells were used as controls. All *in vitro* experiments were performed on microplates with at least four parallel wells.

Ursolic and oleanolic acid were dissolved in dimethyl sulfoxide (DMSO; Sigma-Aldrich, Ayrshire, UK) and stored at 2–8 °C as stock solutions. For all experiments, final concentrations of the tested compounds were prepared by diluting the stock solution with DMEM. The highest DMSO concentration (0.1%) of the medium did not have any significant effect on cell proliferation. The dilution rate was 1:1000, and the concentration of stock solution was 10 mM.

Cell viability was calculated using the formula:

{[(ε_OX_)λ_2_ Aλ_1_ − (ε_OX_)λ_1_ Aλ_2_ of test agent dilution]/[(ε_OX_)λ_2_ A°λ_1_ − (ε_OX_)λ_1_ A°λ_2_ of untreated positive growth control]} × 100

where ε_OX_ = molar extinction coefficient of alamar Blue oxidized form (BLUE); A = absorbance of test wells; A° = absorbance of positive growth control well (cells without tested compounds); λ_1_= 570 nm and λ_2_ = 600 nm

The coefficient of drug interaction (CDI) was used to analyze the interactions between the pure compounds while used as mixture, with or without cyclodextrin complexation; according to CDI values, the interactions were categorized synergism, additivity or antagonism, respectively. CDI was calculated as follows: CDI = AB/(A × B) where:

AB = absorbancy value for the mixture of the two active agents/absorbancy value for the control

A and B = absorbancy value for the single active agent / absorbancy value for the control.

A CDI value <1, =1 or >1 indicates that the drugs are synergistic, additive or antagonistic, respectively. A CDI value less than 0.7 indicates that the drugs are significantly synergistic [46,47].

### 3.2. Preparation of Inclusion Complexes

The preparation of inclusion complexes was already described in detail in our previous papers [15,17]. Briefly, OA and UA, respectively, and HPGCD were kneaded with a 50% ethanol solution in quantities corresponding to a molar ratio of 1:1 triterpene: CD (M_UA_ = M_OA_ = 456.7; M_HPGCD_ = 1761.76). The mixture of UA and OA was prepared as 1:1 molar ratio; its inclusion complex with HPGCD was prepared using the same kneading procedure, in final molar ratio of 0.5:0.5:1 (UA:OA:HPGCD).

### 3.3. In Vivo Experimental Cancer Procedure

SKH1 females, 8 weeks old mice were obtained from Charles River Germany and divided in four groups (six mice/group): group 1 (used as control)—mice were exposed to UVB and 7,12-dimethylbenz(a)anthracene (DMBA) (390 nmol/0.1 mL acetone) was topically applied on the back skin (a single application in the first week of experiment) before irradiation; groups 2, 3 and 4 were treated with 200 μL of 2% aqueous solutions of OA:HPGCD, UA:HPGCD and OA/UA:HPGCD, respectively, 1/2 h before application of carcinogens [36,48]. For UVB exposure, cages were placed in an automatically time-switched irradiation setup. In the experiment, VL-6.M/6W (312 nm wavelength and 680 μW/cm2 intensity at 15 cm) tubes (VilberLourmat, Torcy, France) were used. Under the lamps the minimal erythema dose (MED) of hairless SKH-1 mice, was ≈300 J/m2 [17]. The exposure protocol was the following: irradiation 5 min / day, 2 times/week for 6 weeks, total dose being around 200 J/m2 UVB radiation. During exposure the mice were maintained in a plastic cage and the distance between the lamp and the back of the mice was 15 cm [36].

### 3.4. Non-Invasive Skin Measurements

The following skin parameters were evaluated using a Courage-Khazaka multiprobe adapter, MPA-5 (Cologne, Germany): transepidermal water loss (TWL) using Tewameter^®^TM 300 probe, skin-pH using the Skin-pH-Meter^®^PH 905 probe, sebum using the Sebumeter^®^SM 815 probe, melanin and erythema using a Mexameter^®^MX 18 probe and *stratum corneum* (SC) moisture content using a Corneometer^®^CM 825 probe. Melanin and erythema values were spectrophotometrically determined at 2 wavelengths, respectively: 660 and 880 nm for melanin and 560 and 660 nm for erytema [17,49]. The measurements were conducted every three days after radiation exposure, on a 5 mm diameter back area of the mouse.

### 3.5. Statistical Analysis

All data were analyzed using paired Student’s *t* tests or One-way Anova followed by Bonferroni’s post-tests in order to establish the statistical difference between experimental and control groups; *, ** and *** indicate *p* < 0.05, *p* < 0.01 and *p* < 0.001. A 0.05 level of probability was taken as level of significance.

### 3.6. Compliance with Ethics Requirements

Authors declare that they have no conflict of interest and all procedures involving animal subjects complied with the specific regulations and standards. The experiment was first evaluated and approved by the Ethical Committee of the “Victor Babes” University of Medicine and Pharmacy Timisoara, Romania. The work protocol followed the rules of National Institute of Animal Health: throughout the experiment animals were maintained under standard conditions: 12 h light-dark cycle, food and water *ad libitum*, temperature 24 ± 1 °C, and humidity above 55%. At the end of the experiment, animals were sacrificed by cervical dislocation.

## 4. Conclusions

The synergistic *in vitro* activity of oleanolic and ursolic acids was evaluated on human melanoma cell lines revealing the capacity of the two active agents to potentiate each other’s antiproliferative activity. Hydroxypropyl-γ-cyclodextrin was chosen as a water soluble carrier for these triterpenic acids as well as their mixture in order to be used in chemically (DMBA/TPA) and UV induced murine skin cancers. The measurements of transpidermal water loss, erythema, and skin hydration are readily available and of clinical importance; objective, fast and reproducible results were obtained in terms of detecting skin cancers status. The synergistic activity of oleanolic and ursolic acids was also confirmed by the *in vivo* study.

## References

[1] Dewick P.M. (2001). The Mevalonate. and Deoxyxylulose. Phosphate Pathways: Terpenoids. and Steroids. Medicinal Natural Products: A Biosynthetic Approach.

[2] Ferreira D.S., Esperandim V.R., Marçal M.G., Neres N.B.D.R., Cunha N.L., Andrade e Silva M.L., Cunha W.R. (2013). Natural products and Chagas’ disease: The action of triterpenes acids isolated from *Miconia* species. Universitas. Scientiarum..

[3] Kong L., Li S., Liao Q., Zhang Y., Sun R., Zhu X., Zhang Q., Wang J., Wu X., Fang X. (2013). Oleanolic acid and ursolic acid: Novel hepatitis C virus antivirals that inhibit NS5B activity. Antivir. Res..

[4] Huang D., Ding Y., Lia Y., Zhang W.M., Fang W.S., Chen X.G. (2006). Anti-tumor activity of a 3-oxo derivative of oleanolic acid. Cancer Lett..

[5] Liu J. (1995). Pharmacology of oleanolic acid and ursolic acid. J. Ethnopharmacol..

[6] Takada K., Nakane T., Masuda K., Ishii H. (2010). Ursolic acid and oleanolic acid, members of pentacyclic triterpenoid acids, suppress TNF-α-induced E-selectin expression by cultured umbilical vein endothelial cells. Phytomedicine.

[7] Yan S.L., Huang C.Y., Wu S.T., Yin M.C. (2010). Oleanolic acid and ursolic acid induce apoptosis in four human liver cancer cell lines. Toxicol in Vitro.

[8] Ovesná Z., Kozics K., Slamenová D. (2006). Protective effects of ursolic acid and oleanolic acid in leukemic cells. Mutat. Res..

[9] Jiménez-Arellanes A., Luna-Herrera J., Cornejo-Garrido J., López-García S., Castro-Mussot M.E., Meckes-Fischer M., Mata-Espinosa D., Marquina B., Torres J., Hernández-Pando R. (2013). Ursolic and oleanolic acids as antimicrobial and immunomodulatory compounds for tuberculosis treatment. BMC Complement. Altern. Med..

[10] Musabayane C.T., Tufts M.A., Mapanga R.F. (2010). Synergistic antihyperglycemic effects between plant-derived oleanolic acid and insulin in streptozotocin-induced diabetic rats. Ren. Fail..

[11] Furtado R.A., Rodrigues E.P., Araújo F.R., Oliveira W.L., Furtado M.A., Castro M.B., Cunha W.R., Tavares D.C. (2008). Ursolic acid and oleanolic acid suppress preneoplastic lesions induced by 1,2-dimethylhydrazine in rat colon. Toxicol. Pathol..

[12] Pott P. (1975). Chirurgical Observations Relative to the Cancer of the Scrotum.

[13] Poirier M.C., Beland F.A. (1997). Aromatic amine-DNA adduct formation in chronically exposed mice: considerations for human comparison. Mutat. Res..

[14] Weston A., Harris C.C., Bast R.C., Kufe D.W., Pollock R.E., Weichselbaum R.R., Holland J.F., Frei E. (2000). Chemical Carcinogenesis. Holland-FreiCancer Medicine.

[15] Cerga (Vlaston) O., Borcan F., Bernad E., Popovici I. (2012). *In vivo* evaluation of cyclodextrin complexes with oleanolic and ursolic acids. J. Agroaliment. Process. Technol..

[16] Wang H.M., Soica C.M., Wenz G. (2012). A comparison investigation on the solubilization of betulin and betulinic acid in cyclodextrin derivatives. Nat. Prod. Comm..

[17] Cerga O., Borcan F., Ambrus R., Popovici I. (2011). Syntheses of new cyclodextrin complexes with oleanolic and ursolic acids. J. Agroaliment. Process. Technol..

[18] Sass C., Bojin F., Heges A., Galuscan A., Paunescu V. (2012). Oleanolic and Ursolic Acid in Human Skin Cancer—A Preliminary *in vitro* comparative study. Fiziol. Physiol..

[19] Cerga O., Borcan F., Sass C., Galuscan A., Popovici I. (2012). *In Vitro* Activity Of Ursolic and Oleanolic Acid On A2058 (Human Melanoma) And A2780 (Hepatic Carcinoma). Med. Evol..

[20] Andersson D., Liu J.J., Nilsson A., Duan R.D. (2003). Ursolic acid inhibits proliferation and stimulates apoptosis in HT29 cells following activation of alkaline sphingomyelinase. Anticancer Res..

[21] Es-saady D., Simon A., Ollier M., Maurizis J.C., Chulia A.J., Delage C. (1996). Inhibitory effect of ursolic acid on B16 proliferation through cell cycle arrest. Cancer Lett..

[22] Hsu Y.L., Kuo P.L., Lin C.C. (2004). Proliferative inhibition, cell-cycle dysregulation, and induction of apoptosis by ursolic acid in human non-small cell lung cancer A549 cells. Life Sci..

[23] Wang J., Liu L., Qiu H., Zhang X., Guo W., Chen W., Tian Y., Fu L., Dingbo S., Cheng J. (2013). Ursolic acid simultaneously targets multiple signaling pathways to suppress proliferation and induce apoptosis in colon cancer cells. PLoS One.

[24] Juan M.E., Planas J.M., Ruiz-Gutierrez V., Daniel H., Wenzel U. (2008). Antiproliferative and apoptosis-inducing effects of maslinic and oleanolic acids, two pentacyclic triterpenes from olives, on HT-29 colon cancer cells. Br. J. Nutr..

[25] Shyu M.H., Kao T.C., Yen G.C. (2010). Oleanolic Acid and Ursolic Acid Induce Apoptosis in HuH7 Human Hepatocellular Carcinoma Cells through a Mitochondrial-Dependent Pathway and Downregulation of XIAP. J. Agric. Food Chem..

[26] Li H., He N., Li X., Zhou L., Zhao M., Jiang H., Zhang X. (2013). Oleanolic acid inhibits proliferation and induces apoptosis in NB4 cells by targeting PML/RARα. Oncol. Lett..

[27] Shan J., Xuan Y., Ruan S., Sun M. (2011). Proliferation-inhibiting and apoptosis-inducing effects of ursolic acid and oleanolic acid on multi-drug resistance cancer cells *in vitro*. Chin. J. Integr. Med..

[28] Yadav V.R., Prasad S., Kannappan R., Ravindran J., Chaturvedi M.M., Vaahtera L., Parkkinen J., Aggarwal B.B. (2010). Cyclodextrin-complexed curcumin exhibits anti-inflammatory and antiproliferative activities superior to those of curcumin through higher cellular uptake. Biochem. Pharmacol..

[29] Mendonça E.A., Lira M.C., Rabello M.M., Cavalcanti I.M., Galdino S.L., Pitta I.R., Lima Mdo C., Pitta M.G., Hernandes M.Z., Santos-Magalhães N.S. (2012). Enhanced antiproliferative activity of the new anticancer candidate LPSF/AC04 in cyclodextrin inclusion complexes encapsulated into liposomes. AAPS PharmSciTech.

[30] Danciu C., Soica C., Oltean M., Avram S., Borcan F., Csanyi E., Ambrus R., Zupko I., Muntean D., Dehelean C.A. (2014). Genistein in 1:1 inclusion complexes with ramified cyclodextrins: Theoretical, physicochemical and biological evaluation. Int. J. Mol. Sci..

[31] Dreassi E., Zizzari A.T., Mori M., Filippi I., Belfiore A., Naldini A., Carraro F., Santucci A., Schenone S., Botta M. (2010). 2-Hydroxypropyl-β-cyclodextrin strongly improves water solubility and anti-proliferative activity of pyrazolo[3,4-d]pyrimidines Src-Abl dual inhibitors. Eur. J. Med. Chem..

[32] Pourgholami M.H., Wangoo K.T., Morris D.L. (2008). Albendazole-cyclodextrin complex: Enhanced cytotoxicity in ovarian cancer cells. Anticancer Res..

[33] Soica C., Dehelean C., Danciu C., Wang H.M., Wenz G., Ambrus R., Bojin F., Anghel M. (2012). Betulin Complex in γ-Cyclodextrin Derivatives: Properties and antineoplasic activities in *in vitro* and *in vivo* tumor models. Int. J. Mol. Sci..

[34] Buriez O., Heldt J.M., Labbé E., Vessières A., Jaouen G., Amatore C. (2008). Reactivity and antiproliferative activity of ferrocenyl-tamoxifen adducts with cyclodextrins against hormone-independent breast-cancer cell lines. Chemistry.

[35] Hipler U.C., Schönfelder U., Hipler C., Elsner P. (2007). Influence of cyclodextrins on the proliferation of HaCaT keratinocytes *in vitro*. J. Biomed. Mater. Res. Part A.

[36] Ciurlea S., Bojin M.F., Csanyi E., Ionescu I., Borcan F., Galuscan A., Dehelean C.A. (2011). Evaluation of skin parameters changes in chemical and photochemical initiated tumors on SKH1 mice. Fiziol. Physiol..

[37] McNeil E.M., Ritchie A.M., Melton D.W. (2013). The toxicity of nitrofuran compounds on melanoma and neuroblastoma cells is enhanced by Olaparib and ameliorated by melanin pigment. DNA Repair (Amst).

[38] Swalwell H., Latimer J., Haywood R.M., Birch-Machin M.A. (2012). Investigating the role of melanin in UVA/UVB- and hydrogen peroxide-induced cellular and mitochondrial ROS production and mitochondrial DNA damage in human melanoma cells. Free Radic. Biol. Med..

[39] Liu J., Man W.Y., Lv C.Z., Song S.P., Shi Y.J., Elias P.M., Man M.Q. (2010). Epidermal permeability barrier recovery is delayed in vitiligo-involved sites. Skin Pharmacol. Physiol..

[40] Gunathilake R., Schurer N.Y., Shoo B.A., Celli A., Hachem J.P., Crumrine D., Sirimanna G., Feingold K.R., Mauro T.M., Elias P.M. (2009). pH-regulated mechanisms account for pigment-type differences in epidermal barrier function. J. Invest. Dermatol..

[41] Hachem J.P., Crumrine D., Fluhr J., Brown B.E., Feingold K.R., Elias P.M. (2003). pH directly regulates epidermal permeability barrier homeostasis, and stratum corneum integrity/cohesion. J. Invest. Dermatol..

[42] Tagami H. (2008). Functional characteristics of the stratum corneum in photoaged skin in comparison with those found in intrinsic aging. Arch. Dermatol. Res..

[43] Seité S., Colige A., Piquemal-Vivenot P., Montastier C., Fourtanier A., Lapière C., Nusgens B. (2000). A full-UV spectrum absorbing daily use cream protects human skin against biological changes occurring in photoaging. Photodermatol. Photoimmunol. Photomed..

[44] Scimeca J.V., Zimmerman A.C., Mettler M.F., Kudo A., Kawasaki Y. (2010). Cosmetic Treatment System and Methods. U.S. Patent.

[45] Farwick M., Köhler T., Schild J., Mentel M., Maczkiewitz U., Pagani V., Bonfigli A., Rigano L., Bureik D., Gauglitz G.G. (2014). Pentacyclic triterpenes from *Terminalia arjuna* show multiple benefits on aged and dry skin. Skin Pharmacol. Physiol..

[46] Hao J.Q., Li Q., Xu S.P., Shen Y.X., Sun G.Y. (2008). Effect of lumiracoxib on proliferation and apoptosis of human nonsmall cell lung cancer cells *in vitro*. Chin. Med. J. (Engl.).

[47] Zhou X., Zhang Y., Li Y., Hao X., Liu X., Wang Y. (2012). Azithromycin Synergistically Enhances Anti-Proliferative Activity of Vincristine in Cervical and Gastric Cancer Cells. Cancers (Basel).

[48] CyclodextrinKnowledgeBase. http://www.cyclodextrin.net.

[49] Dehelean C.A., Feflea S., Gheorgheosu D., Ganta S., Cimpean A.M., Muntean D., Amiji M.M. (2013). Anti-angiogenic and anti-cancer evaluation of betulin nanoemulsion in chicken chorioallantoic membrane and skin carcinoma in balb/c mice. J. Biomed. Nanotechnol..

